# User-Adapted Web Services by Extending the eIDAS Specification with Functional Attributes

**DOI:** 10.3390/ijerph18083980

**Published:** 2021-04-09

**Authors:** Lourdes Marco, Alejandro Pozo, Gabriel Huecas, Juan Quemada, Álvaro Alonso

**Affiliations:** Escuela Técnica Superior de Ingenieros de Telecomunicación, Universidad Politécnica de Madrid, 28040 Madrid, Spain; alejandro.pozo@upm.es (A.P.); gabriel.huecas@upm.es (G.H.); juan.quemada@upm.es (J.Q.); alvaro.alonso@upm.es (Á.A.)

**Keywords:** functional attributes, electronic identification, eIDAS, digital accessibility, identity, e-services

## Abstract

To provide web services adapted to the users’ functional capabilities, diversity must be considered from the conceptualization and design phases of the services’ development. In previous work, we proposed a model that allows the provisioning of adapted interfaces based on users’ identity and their functional attributes to facilitate this task for software designers and developers. However, these identities and attributes are self-declared by the users, which may impact reliability and usability. In this work, we propose an extension of our model to resolve these deficiencies by delegating the identity and attributes’ provision to external certified entities. The European electronic Identification, Authentication and Trust Services (eIDAS) regulation established a solution to ensure the cross-border mutual recognition of Electronic Identification (eID) mechanisms among the European Member States. This research aims to provide an extension of this regulation mentioned above (eIDAS) to support functional attributes and connect our previously proposed model to this extended eIDAS network. Thanks to this proposal, web services can guarantee adapted and personalized interfaces while improving the functionalities offered without any previous configuration by users and, in a reliable way, since the functional attributes belong to the users’ official eID. As the attribute set provided by eIDAS nodes only contains citizens’ personal and legal ones, we also propose a mechanism to connect the eIDAS network to external attribute providers that could extend the eIDAS profile of users with their functional attributes. We deployed a pilot to validate the proposed model consisting of an identity provider, an eIDAS node supporting the extended reference code, and an attribute provider supporting functional attributes. We also designed and implemented a simple service that supports eID authentication and serves adapted interfaces based on the retrieved extended eIDAS profile. Finally, we developed an experience for getting feedback from a set of real users with different functional capabilities. According to the results, we concluded that the generalized adoption of the proposed solution in the European digital web services will significantly improve their accessibility in terms of ease of use and adaptability to users’ capacities.

## 1. Introduction

Disability involves factors of different natures, which are closely related to body functions and structures, as well as the environment and participation. Explanatory models such as the medical one have often proposed that some of these factors (body functions) are the cause of the others (participation), leading to biased solutions that have limited the real inclusion of all people in ordinary life. The authors of [1] gave a review of the models of disablement. They presented some approaches, including the International Classification of Impairments, Disabilities, and Handicaps (ICIDH) [2], the Union of the Physically Impaired Against Segregation (UPIAS) [3,4], and the International Classification of Functioning and Disability (ICF) [5]. The latter is a review of the previous ICIDH document, adding new ideas such as disablement being understood as an identifiable variation of human functioning. In the study of [1], the authors reflected on the principle of universalism proposed by ICF, which leads to what is termed the biopsychosocial model, according to which disablement is an intrinsic feature of the human condition, not a difference that essentially distinguishes one subpopulation from another.

Although we understand this biopsychosocial model as an effective and genuinely inclusive perspective, the truth is that the data on the barriers that many people have in their daily lives say otherwise. Especially relevant for this publication are studies such as the one in [6], which highlighted the importance of disaggregating data on disability to identify barriers to exclusion better. As far as Information and Communication Technologies (ICTs) are concerned, a European study carried out in [7] showed that Assistive Technology based on ICT (ICT-AT) can improve people’s daily activity. It also can improve the conditions for leading an independent life by facilitating their communication and interpersonal relationships. They pointed out that the Internet, in particular, has proven extremely useful for end-users. However, the same study also found that education systems were not meeting the needs of students with disabilities, and even educators highlighted that it is essential to develop individualized ICT-AT learning paths.

Within the European Disability Strategy 2010–2020 [8], there is a key commitment to ensure accessibility to goods, services, including public services, and assistive devices for people with disabilities, and making progress on this issue at the European level is seen as a precondition for participation in society and the economy. Nevertheless, on average, in the EU-27, just 5% of the public web services fully comply with the published standards on web accessibility.

The European Union is making significant efforts to stimulate and sustain European Digital Service Infrastructures (DSIs). An example is the eIDAS regulation, which faces privacy and security problems by guaranteeing the reciprocal cross-border recognition of eIDs. The Connecting Europe Facility (CEF) started in 2015 the standardization and specification of the requirements for making eIDAS nodes inseparables. Thanks to this interoperability, European citizens can consume e-services in any Member State by using their national eIDs.

In the last year, solutions such as those mentioned above are gaining particular relevance on the global scene since, in January 2020, the World Health Organization (WHO) declared the outbreak of the novel Coronavirus Disease (COVID-19) to be a Public Health Emergency of International Concern (PHEIC) due to the speed and scale of transmission. This circumstance has triggered a social distancing situation that has affected our system’s spheres, accelerating digital transformation and leading the public and private sphere to establish e-work and e-learning mechanisms where ICTs are the only support. As WHO stated: a certain population, such as those with disability, may be impacted more significantly by COVID-19 [9].

In a previous work [10], we proposed an OAuth2.0 standard-based model for enabling inclusive contexts where users can define their functional attributes in a centralized way. Based on these attributes, services can provide interfaces adjusted to the users’ identity and capabilities. In the proposal, we also presented a list of functional attributes that a person can hold and that corresponds to different interfaces to be provided by services. However, in our previous proposal, the functional attributes were self-declared by users, which could present issues in the reliability of services. The proposal also presented a solution around a centralized identification system (Identity Manager (IdM)), which manages both functional attributes and (email and password-based) credentials. Although this system allowed us to validate the proposed model, many critical factors such as security, reliability, and trust around the request for functional attributes and user identity validation were compromised.

This research aims to propose an extension of the eIDAS specification to include functional attributes and connect our previously proposed model to this extended eIDAS network. Moreover, we propose an architecture to integrate Attribute Providers into the eIDAS network. These Attribute Providers extend the basic eIDAS profile typically provided by the Member States with functional attributes. Thanks to this proposal, web services can guarantee adapted and personalized interfaces while improving the functionalities offered without any previous configuration by users and, in a reliable way, since the functional attributes belong to the users’ official eID.

We provide the required details on how to modify the eIDAS code to include the functional attributes mentioned above so that the other Member States can apply the same procedure to implement the proposed solution. Besides, we deployed the necessary architecture to make this solution compatible with the Spanish eIDAS infrastructure’s specific case. To validate our proposal, we implemented a simple pilot web service consisting of an identity provider, an eIDAS node supporting the extended reference code, and an attribute provider supporting functional attributes. We also tested the proposed solution with real users with different functional capabilities to get their feedback about the experience.

The document is structured according to the following scheme. In Section 2, we analyze the the current related works in the literature. Section 3 describes the extension to eIDAS we propose for supporting functional attributes and connecting the eIDAS nodes to components providing attributes. Section 4 and Section 5 provide the methodology and results, respectively. Finally, Section 6 concludes the work and suggests future lines of research.

## 2. Related Work

Digital identity is the representation of an entity (or group of entities) in the form of one or more information elements that allow the entity (or entities) to be uniquely recognized within a context to the extent that is necessary (for the relevant applications) [11]. At present, most web service providers use registration systems and later user/password systems to manage their users’ identity management. This type of system has several drawbacks for users, as they are forced to remember several different credentials. They are not very reliable for service providers since the provided identity profiles are not verified. Federated Identity Management (FIM) solutions address these identity management problems. As stated in [12], under an FIM, individuals can use the same user name, password, or other personal identification to sign in to the networks of more than one enterprise in order to conduct transactions. The services’ cost is the verification of the credentials presented, and in this way, the users do not need to prove their identity in each of the actions or transactions carried out. It will be enough to have been authenticated by a trusted authority or institution [13].

Currently, in a globalized world where physical frontiers are increasingly blurred, FIM solutions for enabling cross-border authentication mechanisms for citizens when accessing public and private services are becoming more useful every day. In this scope, in many EU Member States, national ID systems rely on eID cards and digital certificates. To support all these different systems, it would be necessary to develop an infrastructure capable of crossing Europe. On more than one occasion, the EU has made great efforts to move towards viable and robust solutions for such an infrastructure. For instance, the EU has financed projects such as STORK (Secure idenTity acrOss boRders linKed), STORK 2.0, or FutureID, or even the eIDAS regulation. In the literature, many studies have described the STORK and FutureID projects [14,15,16,17]. Thanks to both of them and according to the results of these studies, national eIDs can be used for providing cross-border authentication in e-services provided by the Member States.

The project STORK mentioned above laid the foundation for creating a pan-European system that gave the users two identification possibilities. On the one hand, they can communicate directly with the other Member States’ systems using middlewares. On the other hand, they can connect through a common proxy service that acts as an intermediary through the local service providers. STORK 2.0 was the follow-up to the STORK project, and in its development, the foundations of the previous project were laid to apply the designed system in additional use cases. For its part, FutureID was a project based on the conclusions of STORK and STORK 2.0 that also included the application of credentials based on attributes.

On the basis of the European projects mentioned above, in early 2014, the representatives of the Commission, the Council, and the European Parliament (MEP) established a political agreement for a regulation on electronic IDentification and Authentication Services (eIDAS) [18]. This document expresses that the aim of this Regulation is to ensure that for access to cross-border online services offered by Member States, secure electronic identification and authentication are possible. Throughout the text, the minimum requirements with which the Member States must comply to achieve effective integration of services are set out, such as the importance of building technology-neutral solutions. In this respect, the Member States can choose their technological solution to connect services to their eIDAS node, while the eIDAS nodes are always interconnected thanks to the use of the SAML2.0 standard [19]. Although the standard may imply limitations in the integration of eIDAS nodes, some authors of this article showed in [20] that standing out from SAML 2.0, the OAuth 2.0 protocol [21] for delegated authentication can be a simple, scalable, easy integration, and light solution. They proposed a single login point based on a gateway deployed between service providers and the nodes deployed by eIDAS. This gateway is in charge of translating the simple OAuth 2.0 flows produced by the services to the SAML requests that eIDAS nodes require.

Since the eIDAS regulation came into force, it has been supported by European funds through the CEF Telecom program, which has resulted in several projects to integrate eIDAS-compliant eID authentication into web services. In this scope, the main concerns at the time of integrating e-services for authenticating users in the eIDAS infrastructure are: (1) the identification of attributes for each specific domain and (2) the way in which e-services take advantage of those attributes. In this sense, in the literature, many proposals aimed to extend the support of eIDAS attributes, including the definition of attributes with very different uses in very diverse environments such as academia, e-health, or e-banking.

In the academic field, for instance, several studies such as [22,23,24,25,26] showed that the integration of eID authentication can undoubtedly improve the users’ experience and that the definition of new attributes can enhance higher education institutions’ services by exploiting students’ academic profiles.

On the other hand, in the field of health, proposals such as the one carried out in [27], show how the eIDAS node has been extended (in a project called eSENS) for the definition of additional attributes that allow the transfer of patient identifier information. Other approaches such as those conducted in [28] revealed that e-prescriptions might have different benefits such as economic benefits: in terms of efficiency gains, reduction of fraud, or paper printing; health benefits, in terms of reduced error rates or better accessibility to medication; and social benefits, such as patient satisfaction with the health system. All these benefits prove that eIDAS-compliant solutions will improve digital health systems significantly.

The authors in [29,30] investigated fields such as e-banking, analyzing current threats and the adaption of the eIDAS standard towards trusted banking transactions. Specifically, the work of [30] extended the eIDAS definition to biometrically authenticated transactions. The authors identified eIDAS as highly suitable for banking transactions due to its security protocols and infrastructure.

Our review of the current literature specifically focused on implementing eIDAS regulation linked to web inclusion or accessibility, and we found no publications. However, some works have explored [31] or analyzed [32,33] exciting ways to break the barriers of e-Administration. In particular, to avoid complexity in the configuration and access to applications based on digital certificates, the authors of [31] proposed developing a USB device that includes a cryptographic token.

## 3. Proposed Solution

Having analyzed the literature on proposals for implementing eIDAS regulation for inclusive purposes, we can conclude that there is much work to be done. Therefore, it is necessary to continue working along these lines to propose solutions that make it easier for developers and designers to create interfaces adapted to users’ needs.

We propose a solution in this section that enables the use of functional attributes in eIDAS-based services. Thanks to our proposal, web services can improve the functionalities offered without the need to perform any previous configuration while guaranteeing adapted and personalized interfaces for the users.

For enabling available functional attributes for web services connecting to eIDAS, we face two significant challenges. The first one is to extend the eIDAS specification, which, as we will see in the next section, is currently supporting by default only a set of legal and personal attributes. The second challenge consists of including connections to external entities providing attributes in the authentication flow since the Minimum Data Set (MDS) provided by the Identity Providers (IdPs) of the Member States only includes legal and personal attributes.

### 3.1. EIDAS Basis

Before presenting our proposal, in the following paragraphs, we give a brief introduction to understand the basis of personal attributes’ functioning in the eIDAS specification.

In their own words, to support the Digital Single Market in its success the Connecting Europe Facility (CEF) program is funding a set of generic and reusable Digital Service Infrastructures (DSI), also known as building blocks [34]. For each building block, the CEF funding covers a Core Service Platform, which typically includes technical specifications, the source code, and supporting e-services. On the other hand, during the last few years, the CEF has been promoting periodic calls for founding research and development activities with the objective of exploiting the advantages of the building blocks.

The CEF program has released ten building blocks, among which is the eID. Its primary purpose is to allow public administrations and private service providers to authenticate foreign citizens using their eID. These services must comply with the eIDAS regulation in terms of interoperability, trust, and security.

As far as interoperability is concerned, an eIDAS-compliant service enables people or businesses to use their national eID scheme to gain access to services from the other Member States. On the other hand, trust refers to providing and ensuring the legal validity of transactions across borders and the same legal status as traditional paper-based processes. Finally, security is the Levels of Assurance (LoAs) of the eID schemes. Under eIDAS, there is a lower risk of identity theft and misuse of personal information.

**Figure 1:**
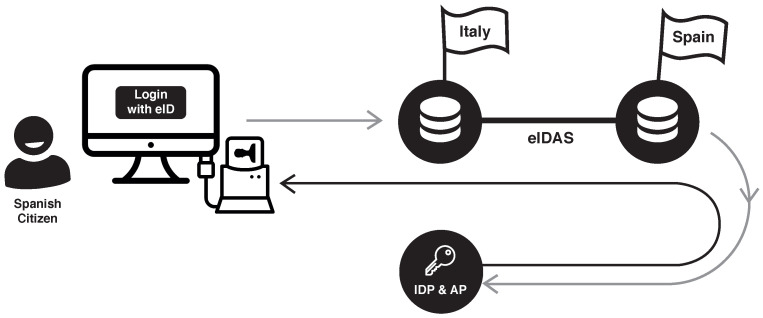
eIDAS basis example.

As can be concluded from the above definitions, public administrations or private service providers can authenticate citizens from any Member State using their national eID. Figure 1 illustrates an example of the process. A citizen from Spain who wants to access a service deployed in Italy is redirected from the Italian eIDAS node to the Spanish eIDAS node to perform the authentication process. Then, the citizen’s identity is verified by the Spanish identity and attribute providers. Finally, the verified request is sent back to the Italian service, allowing the Spanish citizen to access the service. The delegation from one country to another relies on the eIDAS SAML 2.0-based specification, which connects the Member States’ eIDAS nodes.

Beyond mutual recognition of national eID schemes, defining a set of attributes common to the different Member States would make communication between nodes universal, regardless of the authentication scheme notified by each Member State (e.g., certificates or identification cards). There are two types of attributes, (1) natural and (2) legal person. Table 1 shows the list of attributes that the eIDAS specification supports grouped by type. The ones with an asterisk are mandatory in any request to the eIDAS nodes.

Any request from service providers to the eIDAS nodes must include the MDS. The MDS attributes are PersonIdentifier, FamilyName, FirstName, DateOfBirth (from natural person), and LegalPersonIdentifier and LegalName (from legal person). However, as stated before, we detected an increasing need to extend the profile to support functional attributes. Thanks to this, web services can guarantee adapted and personalized interfaces. Therefore, the domain-specific attributes’ integration into the eIDAS infrastructure is essential. Below, we describe how to integrate functional attributes in the eIDAS node.

### 3.2. eIDAS Extension to Support Functional Attributes

As stated above, the eIDAS specification defines natural and legal person attributes (some of them mandatory), but this is still not enough to support the user’s functional capabilities. Therefore, we propose an extension of the eIDAS user profile based on the attributes presented in our previous work [10] and summarized in Table 2.

As we can note, each functional attribute does not unavoidably match with a disability caused by a particular disease, nor with an associated interface or assistive technology. For instance, we may consider a blind person that requires help for using ICTs without vision. In this case, the attribute vision would be defined with a 100% score (meaning a 100% visual disability). However, this does not imply that the service should provide a particular user interface. In this case, it could be a voice interface, but also a text-only interface.

On the other hand, but similarly, we can assess the case of an older person who has Parkinson’s disease. This person has a severe tremor and probably requires assistance for using ICTs with limited manipulation. In this scenario, the attribute manipulation would be defined in the system with an 80% score (meaning an 80% motor disability). This could involve using a voice interface as above, although the disease is quite different.

To define the list of functional attributes to extend the eIDAS MDS, we relied on our previous study [10]. Table 3 summarizes the list of attributes. We define these attributes as the natural person type. We created a new namespace a11y (accessibility) with URL http://eidas.europa.eu/attributes/sectorspecific/a11y (accessed on 3 March 2021).

We modified the eIDAS implementation in order to include the new functional attributes. The code is available on GitHub at the repository https://github.com/aalonsog/eIDAS-node (accessed on 3 March 2021) under the European Union Public License (EUPL). The changes in the eIDAS code were:

- Defining XSD (XML Schema Definition) schemes for the new functional attributes. The a11y_commons/src/main/resources/schema/a11y/ (accessed on 3 March 2021) directory stores said schemes.
- A sample configuration for the new version of the eIDAS components was elaborated based on the eIDAS sample implementation’s sample configuration. The EIDAS-Config-a11y (accessed on 3 March 2021) directory stores said configuration. Besides the sample configuration, the server/idp/user.properties (accessed on 3 March 2021) file contains examples of definitions for all the new functional attributes.
- The functional attributes were added to the saml-engine-additional-attributes* (accessed on 3 March 2021) files placed in the EIDAS-Config-a11y/server (accessed on 3 March 2021) directory and its subdirectories.
- The files contained in the a11y_commons/src/main/java/a11y/ (accessed on 3 March 2021) directory include the corresponding and developed attribute marshallers.
- We modified the file EIDAS-Node/src/main/resources/eu/eidas/node/package.properties (accessed on 3 March 2021) to add the new attribute names. It is necessary to visualize them in the user interface.

### 3.3. Attribute Providers

E-services are able to send requests to an eIDAS node in order to obtain the users’ functional attributes proposed in the list above as part of the eIDAS scheme extension. However, Member States use IdPs to authenticate citizens on the eIDAS network that usually only provide legal and personal attributes. Consequently, when the user’s home country eIDAS node sends an authentication request to the associated national IdP, this IdP will send back a response including only the MDS attributes.

To enrich the user profiles with the new functional attributes, the infrastructure has to delegate external attribute providers that own such information about users. Therefore, the authentication flow has to be modified to include the connection to this type of provider.

Regarding this issue related to attribute providers, we proposed a solution based on our previously proposed architecture in [24]. We used a proxy that intercepts requests between the eIDAS nodes and the IdPs, and vice versa. Its mission is to request extra attributes of the external attribute providers.

**Figure 2:**
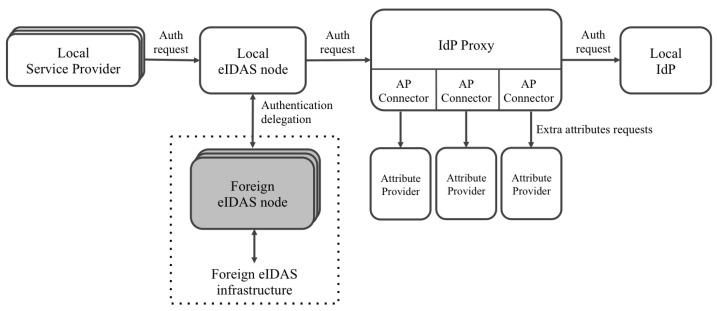
Connection to the attribute providers’ architecture. IdP, Identity Provider.

**Figure 3:**
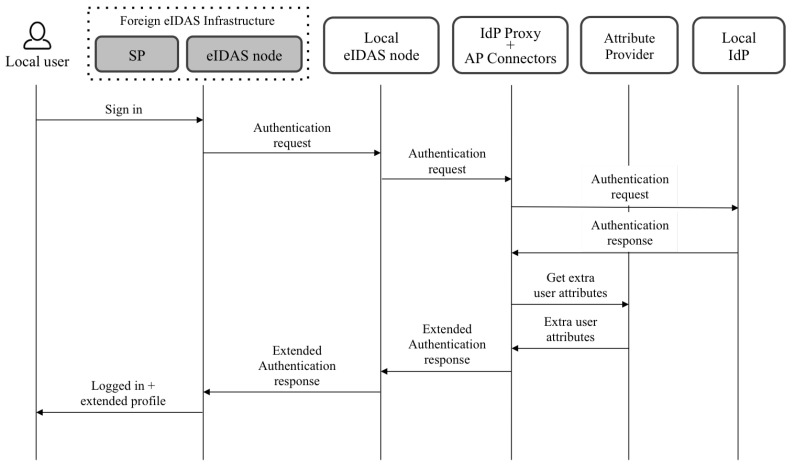
Authentication flow for local users.

**Figure 4:**
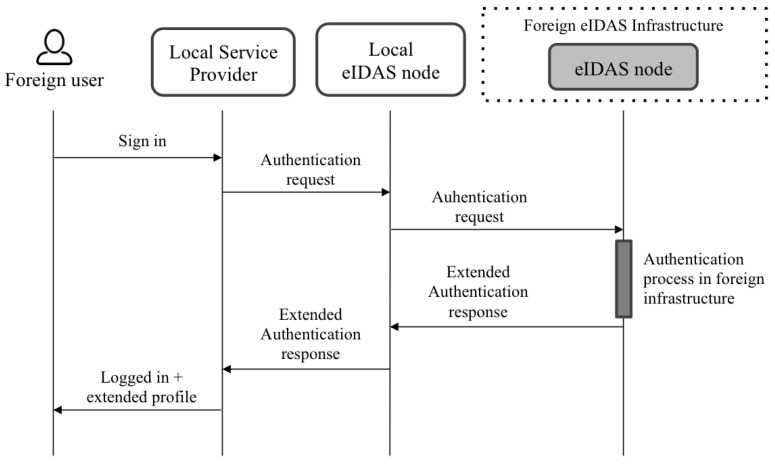
Authentication flow for foreign users.

Figure 2 shows an overview of the architecture, and Figure 3 and Figure 4 show the flows when local and foreign users authenticate in a service provider. The following paragraphs summarize the responsibilities of each component. A detailed description of the architecture and the flows was explained in the referenced work [24].

Users authenticate in service providers that have to be previously registered in the eIDAS node deployed in the country where the service operates. Depending on the users’ nationality, the eIDAS node to which the service is connected redirects the authentication request to the eIDAS node of the users’ country.

Once eIDAS nodes receive authentication requests, they redirect them to the IdP of the corresponding country to proceed with users’ authentication. However, in the proposed architecture, IdP proxies intercept those requests and decrypt them to extract the requested attributes. Once the IdP proxies obtain the attributes, they redirect the requests to the IdP.

eIDAS nodes have to be configured to set the IdP proxy as a callback endpoint for receiving authentication responses from IdPs. Thus, when authentication responses come back to the IdP proxy, it decrypts them again to check which of the requested attributes have been already provided by the IdP. The attributes that have not been provided by the IdP are then requested to the registered attribute providers. The proposal takes into account the possibility of registering several APsfor retrieving the additional attributes. Moreover, each of these APs can support different protocols for accessing the attributes. Thus, a specific connector (named the AP connector) can be developed for each specific AP. After receiving the requested attributes (or a part of them), the IdP proxy includes the extended list in the authentication response, which is encrypted again and sent to the eIDAS nodes to finish the process.

Finally, the eIDAS node sends the response containing the extended eIDAS profile of users to the service provider or the foreign eIDAS node that generated the request.

In this architecture, eIDAS nodes must support the extensions of the eIDAS reference code proposed in this paper to (1) be able to receive authentication requests from service providers requesting new functional attributes and correctly process them and (2) be able to process responses from IdPs and external eIDAS nodes containing new functional attributes and correctly process them.

On the other hand, eIDAS nodes must support connection to IdP proxies. The corresponding SSL certificates have to be registered in the eIDAS nodes, IdP proxies, and IdPs in order to encrypt, decrypt, sign, and validate signed requests and responses.

## 4. Methodology

We deployed a pilot to validate the proposed model consisting of an identity provider, an eIDAS node, and an attribute provider. We also designed and implemented a simple COVID web service that supports eID authentication and serves adapted interfaces based on the retrieved extended eIDAS profile. Finally, we evaluated the proposal by getting feedback from 10 real users with different functional capabilities.

In this section, we provide details about the deployment we carried out, the COVID information service, and the tests’ results with the users.

### 4.1. Implementation and Deployment

We deployed a testing eIDAS node from the extended eIDAS source code we proposed in this paper. The service provider explained in the next subsection was registered in this eIDAS testing node. As this pilot’s objective was to validate the extension of citizens’ profiles with functional attributes and their consumption by adapted e-services, the authentication request was always sent to this single testing eIDAS node, independently of the country of origin selected by the users.

We deployed the testing IdP provided with the eIDAS node in the reference code for authenticating users. This IdP provides authentication to registered users employing a username and password. However, connecting the node to official IdPs deployed by the Member States would present several difficulties. Each Member State defines a set of authentication schemes on its IdP that could make access difficult for people with disabilities. For instance, the Spanish eIDAS node only allows authentication based on the eID using a card reader, which hinders access for people with Parkinson’s disease or blind people. Other countries rely on mobile applications for authentication, but unless these are not accessible, this would present the same problem.

Finally, we developed a demo attribute provider that simulated an official attribute provider’s functionality by answering requests containing the personal identifier of citizens with a JSON containing their functional attributes. The attribute provider sends requests using the HTTP protocol.

To perform the experiment of Section 5 and get the opinion of citizens, we registered in both the IdP and the AP a set of 10 citizens with simulated personal data. However, the AP’s functional attributes corresponded to the citizens’ real functional capabilities that were authenticated with each provided demo user.

**Figure 5:**
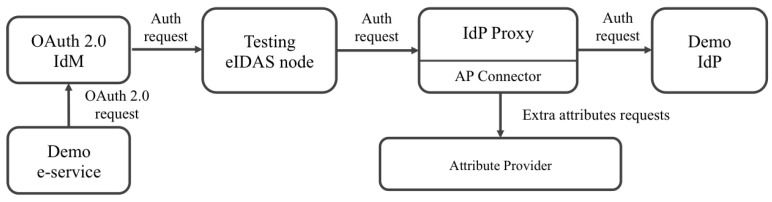
Deployment.

Figure 5 shows the deployed components. As can be observed, the service provider connects to the eIDAS node using the OAuth 2.0 identity manager as a gateway between the OAuth 2.0 and SAML 2.0 protocols. The identity manager also provides a single sign-in feature to the services. The article in [20] described the architecture and technical details of this gateway.

For the sake of simplicity, we deployed all the components in a single virtual machine of an Openstack (Openstack: https://www.openstack.org (accessed on 3 March 2021)) infrastructure with the technical specifications show in Table 4. On the other hand, Table 5 shows the addresses for which each component was deployed. Some of them were public because citizens need open access to them to consume the services or are redirected to them during the authentication process by the web browser. The access to the private ones was limited to components deployed in the same infrastructure. Even being public, some of the listed URLs in Table 5 are not accessible because specific paths or parameters in the requests are needed and set by other components during the authentication process.

### 4.2. Demo e-Service

As mentioned above, we designed and developed a simple COVID web information service that supports eID authentication. It serves adapted interfaces based on the retrieved extended eIDAS profile (source code available at GitHub (https://github.com/Lourdesmarco/oAuth-COVID-service (accessed on 3 March 2021))). Proposed on a pilot basis, the service aims to inform high-risk populations (such as those with a disability) about COVID-19 and also allows them to request services that would involve leaving their homes in a risky situation, such as buying protective materials or requesting a test. The application provides interfaces adapted to the users’ capabilities that are obtained from the functional attributes provided by the infrastructure after the authentication process. Table 6 shows the user interfaces designed for this experiment and the conditions users have to fit for obtaining each of them.

As stated before, we registered in both the IdP and the AP a set of 10 users, including the mandatory eIDAS attributes and a set of functional attributes based on the actual users’ capabilities who took the test. We list the registered users and the attributes defined in Table 7. To simplify the creation of documentation and guides for the users taking part in the test, we set the same password for all users.

**Figure 6:**
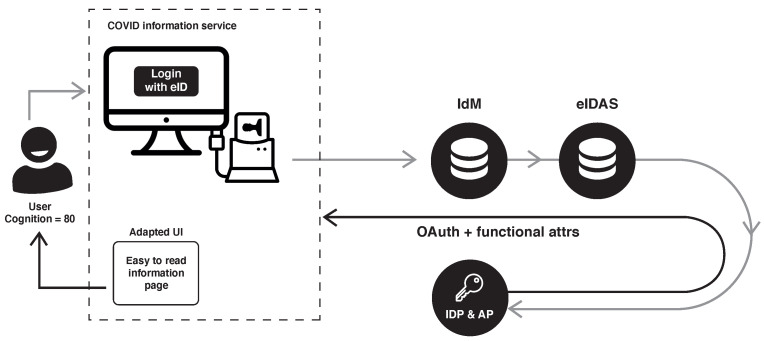
Demo e-service diagram.

Figure 6 shows a diagram that illustrates how a user accesses the COVID information service and how, through eIDAS and the attribute provider, we can get the information specifically adapted to the user’s need. Once the users log in, as their eID validates their credentials, they will have at their disposal services for which their fiscal code needs to be confirmed, such as requesting a Polymerase Chain Reaction (PCR) COVID test.

**Figure 7:**
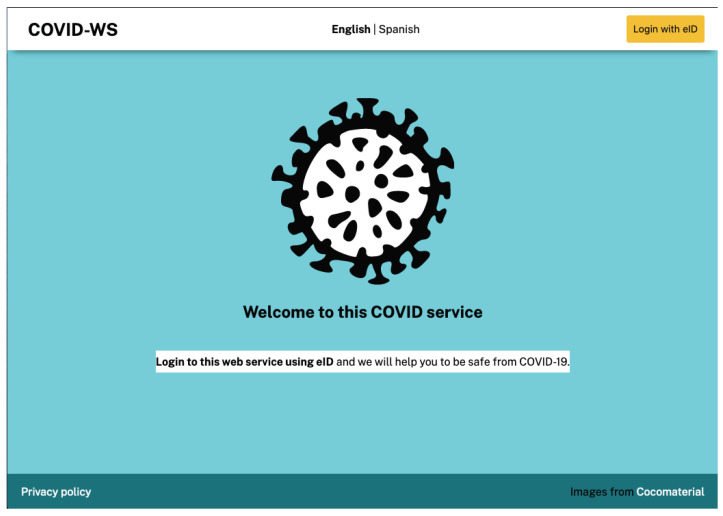
COVID information service login page.

The user in the example has a severe cognition disability, meaning she cannot understand difficult text. To get a custom and adapted experience with the COVID information service and be able to request a test to know if she is infected, she needs to log in by pressing the Login with eID button on the main page (Figure 7).

Once users click on the login button, they are redirected to the OAuth 2.0 IdM service, where they will have to indicate their country of origin. The IdM, as we have explained before, checks if the service is registered and sends an SAML 2.0 request to the country’s node that the user has chosen. As stated before, independent of the selected country of origin, the user is redirected to the same testing eIDAS node, represented as ES (Spanish node).

**Figure 8:**
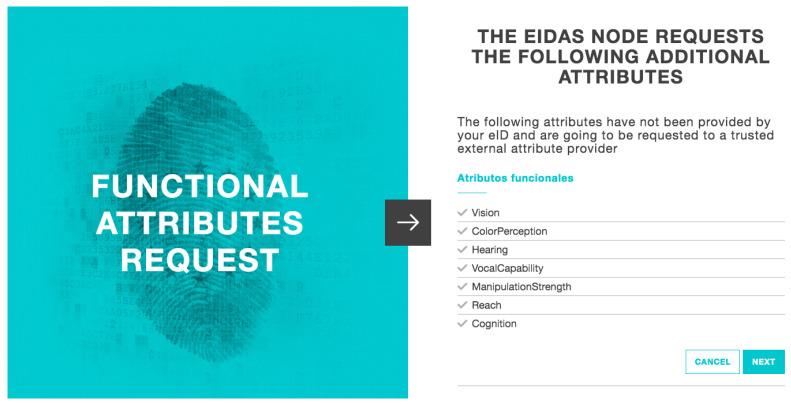
eIDAS node functional attributes request.

After country selection, the next step is to validate the users’ identity in the eIDAS node. With a total of 3 simple views, the users are guided to the introduction of their username and password (data previously registered in the IdP). When the eIDAS node has validated the data, it will return the response with the users’ data from their eID and the attribute provider’s functional attributes (Figure 8).

**Figure 9:**
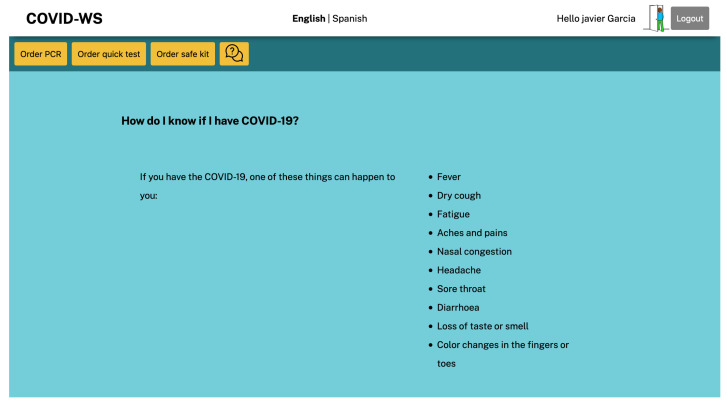
COVID information service adapted to cognition disability.

The COVID information app then receives the eIDAS node’s response, which includes the user’s data and functional attributes. Since this is a service developed with node.js (node.js: https://nodejs.org/ (accessed on 3 March 2021)) and the express.js framework (express.js: https://expressjs.com/ (accessed on 3 March 2021)), the middleware will decide to show the proper User Interface (UI) depending on the attributes linked to their eID. As the user in this example has a cognitive disability, the application logic was coded so that the middleware responds by sending a UI where the information follows easy-to-read conventions (Figure 9).

**Figure 10:**
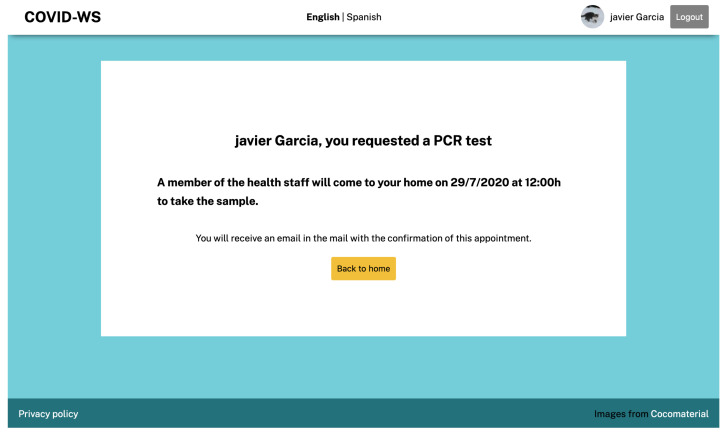
COVID information service confirmation message.

Once the users have accessed the service with their eID, they can make several requests related to COVID-19, such as asking to be tested for the disease or asking for protective materials. Imagine that the user in the example has some symptoms and decides to ask for a PCR test at home. As the service has already validated her identity reliably and securely, the procedure is reduced to clicking on the Order PCR test button and confirming that she wants to make this request. After the request has been confirmed, the user receives a confirmation message with the test’s necessary information (Figure 10).

## 5. Results

To evaluate this research work, as we detailed in the two previous sections, we deployed a test environment consisting of an identity provider, an eIDAS node, an attribute provider, and a simple web service that supports eID authentication. The latter serves adapted interfaces based on the retrieved extended eIDAS profile. Besides, to ensure that this testing environment could be applied to real scenarios, we conducted an experiment in which a group of 10 users had to request a home PCR test using the developed COVID application. At the end of each test, we asked the users their opinion through a survey.

Our aim with the accomplishment of this experiment and the survey was to evaluate two main aspects: (1) whether people with disabilities consuming web services believe that the use of their capabilities improves the accessibility and the user experience of such services and (2) whether people with disabilities notice an improvement in the web services thanks to the integration of their functional attributes in their eID profiles.

Before testing the service, users answered an initial question providing their opinion about the appropriateness of adding information about their functional attributes to their national eIDs. They then followed a guide to authenticate themselves into the service (using their eID) and consumed it. After using the service, the users had to respond to three more questions to determine their opinion about the developed service and if the solution proposed in this paper fits in other web services.

A total of 26 people with different capabilities voluntarily participated in the study. As we show in Table 7, we separated the participants into a total of 4 groups, either because their disability was related to a specific attribute or because they were people with several affected attributes or none at all. The groups were named as follows: (1) visual impairments, (2) hearing impairments, (3) aged people, and (4) no functional attributes defined. Thanks to the collaboration of the deaf community for evaluating the experiment, the amount of users in the group hearing impairments was bigger than the rest of the groups. Table 8 shows the questions and the results of the survey. As noted above, users answered the first question before the test, and after the test, users answered the following three questions.

As we can see from the survey results, most users answered “yes” (96.2%) to the pre-test question (1). We asked them if they believed that including their functional attributes in their eID can improve the usability, accessibility, and quality of web services. In the post-test Questions 2 and 3, eighty-four percent and 91.7% of the users responded that having used an extended eID that includes their capabilities made it easier to meet the website’s objectives and improved its accessibility. In the fourth question, ninety-five-point-eight percent of users responded that they would like web services to include this functionality after using this pilot.

From the results presented, we can conclude that most of the users participating in this experiment thought that using their capabilities through an eID contributed to improving the accessibility and experience with a web service. We can also conclude that most of them noticed a clear improvement of the websites thanks to integrating their functional attributes with their eID profiles.

It is important to note that only one user responded negatively to the survey. After conducting a personal interview, we learned that this was due to a specific problem with the Identity Manager (IdM) that only users who consumed the information with a screen-reader could detect. We must point out that this research work did no examine nor correct the specific accessibility problems that the reused pieces of code from other previous proposals had in their UIs, such as those linked to the eIDAS node or the IdM. We sincerely believe that this was beyond the scope of this work.

Some reasons justify the small number of users who tested the deployed pilot. On the one hand, access to people’s communities with functional diversity is always a challenge since strict rules govern them to protect the people who attend the centers. On the other hand, one of the big problems we continue to fight is the digital divide, which makes it very difficult to directly reach people with functional diversity through, for example, social networks or personal websites. Finally, the exceptional situation of COVID has meant an additional limitation in the access to centers or meetings in which physical presence is necessary.

Finally, Table 9 shows a comparison between the eIDAS-based solution we proposed and other available solutions in the literature that we analyzed in Section 2. As can be observed, given that most of the projects are framed within the eIDAS infrastructure, almost all proposals have developed eID support for user authentication and are cross-border solutions. However, only some of these solutions, such as those proposed by [23,24,27], have extended the eIDAS node attributes in order to be able to incorporate extra user data during the authentication process. The works [23,24] were the only approaches that included support for Federated Identity Management (FIM), and only [31] was presented as a solution to address web accessibility in user identification systems. It is pertinent to note that of those analyzed, the latter was the only one proposed that did not fall under the eIDAS project, so it is not a cross-border solution. Another aspect worth noting is that of the references analyzed, none of them proposed a multi-interface solution based on user attributes.

Like most of the solutions analyzed, ours enables secure cross-border authentication based on the eID through the eIDAS infrastructure. However, the inclusion of the eID supposes some drawbacks from a user experience perspective. The authors of [35] did a survey and concluded that it did not compensate for the eID benefit obtained concerning citizens’ effort to obtain and use the eID. The inclusion of extra attributes in the eID profile allows enriching the services connected to the eIDAS infrastructure and may improve the user experience. In contrast to our solution, not all of the eIDAS-based solutions tackle this issue.

The significant difference between our solution and the cross-border solutions analyzed is that ours contemplates web accessibility. In this regard, it is worth noting that our work lays the foundation for building a software model to facilitate the creation of multi-interface web services. Thanks to this model, e-services are able to obtain users’ functional attributes in order to adapt the provided UIs to the users’ needs. Therefore, we did not directly provide and evaluate accessible UIs, but a mechanism to allow services to have as much information as possible about users’ capabilities in order to design and serve those accessible interfaces. Service designers should follow the indications of ISO 9241 [36] and ISO 25063 [37] to improve UIs’ usability and quality and, then, based on the model proposed in this work, decide which of them serve to users based on their functional attributes.

## 6. Conclusions and Future Work

This research work proposed an extension of the eIDAS regulation to support functional attributes and connect our previously proposed model to this extended eIDAS network. Thanks to this proposal, web services can guarantee adapted and personalized interfaces while improving the functionalities offered without any previous configuration by users and in a reliable way, since the functional attributes belong to the users’ official eID.

Furthermore, this work presented a mechanism to connect the eIDAS network to external attribute providers that could extend the eIDAS profile of users with their functional attributes. Thanks to this, as seen in the example given in this research, third-party institutions of different sectors can provide functional attributes to extend users’ eIDAS profiles dynamically.

We developed and deployed an extended testing eIDAS node as an instance of the proposed solution, including a testing IdP for authenticating users and a demo AP that simulates an official AP’s functionality. We also deployed a simple web service that supports eID authentication and serves adapted interfaces based on the retrieved extended eIDAS profile.

We gathered the opinion of 10 users with different capabilities who tested the service and completed a survey. The feedback received was very positive, with 100% of the users in favor of including this initiative in other web services.

After the proposal validation, it is essential to point out some considerations drawn from this service’s implementation. The main limitation of the developed infrastructure is the eIDAS cross-border feature and the attribute extension. To fully enable a cross-border solution, all Member States where the service is available will have to implement the extension of functional attributes in their eIDAS nodes as described in this research work. However, there is no common approach to managing the spread of attributes, so these scenarios could only happen under agreements between countries. On the other hand, the node’s identification process should be simplified as much as possible, reducing the number of screens and trying to make the process as agile as possible for the user. Likewise, each Member State should define authentication schemes that ensure accessibility and do not hinder the authentication process performed by disabled people.

Concerning future extensions of this work, the possibility of offering advantages or services adapted to capabilities (for instance, discounts on tickets or access to restricted areas) is opened up. Another possible research line may include applying the proposed model to a complex interface and studying how to simplify component-based UIs’ construction. The study of each user group’s specific needs to ensure that the solution is correctly adapted to all circumstances could also be a good way forward.

Finally, further research might explore self-sovereign solutions based on blockchain. Blockchain technology [38] enables users to verify, preserve, and synchronize the contents of a data sheet (a transaction ledger) replicated by multiple users. In this scope, self-sovereign identity-based approaches [39] would allow people to have total control over the functional attributes. In this way, the European Union is developing the European Self-Sovereign Identity Framework (ESSIF) [40], which is compatible with eIDAS, to provide a decentralized infrastructure for European identity management.

Thanks to the connection of our proposed model to the eIDAS network, we can ensure (1) that the users are who they say they are (eID) and (2) that the provided functional attributes are valid (official AP). Furthermore, as we can conclude from the limitations, there is a great need to standardize how attribute providers connect to the nodes, which would result in a common framework for future attribute extensions in any field.

## Figures and Tables

**Table 1:** Available natural and legal person attributes in eIDAS nodes.

Friendly Name	NameUri	Namespace
**Natural person**
PersonIdentifier *	/naturalperson/PersonIdentifier	eidas
FamilyName *	/naturalperson/CurrentFamilyName	eidas
FirstName *	/naturalperson/FirstName	eidas
DateOfBirth *	/naturalperson/DateOfBirth	eidas
BirthName	/naturalperson/BirthName	eidas
PlaceOfBirth	/naturalperson/PlaceOfBirth	eidas
CurrentAddress	/naturalperson/CurrentAddress	eidas
Gender	/naturalperson/Gender	eidas
**Legal person**
LegalPersonIdentifier *	/legalperson/LegalPersonIdentifier	eidas
LegalName *	/legalperson/LegalName	eidas
LegalAddress	/legalperson/LegalPersonAddress	eidas
VATRegistration	/legalperson/VATRegistrationNumber	eidas
TaxReference	/legalperson/TaxReference	eidas
D-2012-17-EUIdentifier	/legalperson/D-2012-17-EUIdentifier	eidas
LEI (Legal Entity Identifier)	/legalperson/LEI	eidas
EORI (Economic Operators Registration and Identification)	/legalperson/EORI	eidas
SEED (System for Exchange of Excise Data)	/legalperson/SEED	eidas
SIC (Standard Industrial Classification)	/legalperson/SIC	eidas

Note: NameUri from http://eidas.europa.eu/attributes (accessed on 3 March 2021). * Mandatory attributes.

**Table 2:** Functional attributes.

Attribute	%	Interface/Feature
Vision	100	Voice User Interface
		Only text
	1–99	Magnification
		Control of contrast
		Reduction of RFV
Color Perception	1–100	No color meaning
		Color-coded (tags)
Hearing	100	Sign language, subtitles
		Tactile user interface
	1–99	Audio clarity
		No background noise
		Increase volume range
Vocal Capability	1–100	Orally-generated sounds
		Keyboard/pen
		Touch user interfaces
Manipulation	1–100	One-handed operations
Strength		Sequential key entry
		Voice user interfaces
Reach	1–100	Target height or position
Cognition	1–100	Simpler
		Easier to use
		Timing, errors, focus

**Table 3:** Functional attributes proposed for extending the eIDAS specification.

Friendly Name	NameUri (http://eidas.europa.eu/attributes (Accessed on 3 March 2021))	Namespace
Vision	/naturalperson/vision	a11y
ColorPerception	/naturalperson/colorperception	a11y
Hearing	/naturalperson/hearing	a11y
VocalCapability	/naturalperson/vocalcapability	a11y
ManipulationStrength	/naturalperson/manipulationstrength	a11y
Reach	/naturalperson/reach	a11y
Cognition	/naturalperson/cognition	a11y

Note: NameUri from http://eidas.europa.eu/attributes/sectorspecific/a11y (accessed on 3 March 2021).

**Table 4:** Openstack instance specifications.

**Openstack flavor**	m1.medium
**CPU**	2 virtual CPU
**Memory**	4 GB
**Disk**	40 GB
**Operating System**	Ubuntu 14.04

**Table 5:** Components’ URLs. IdM, Identity Manager.

Component	URL	Type
Demo e-service	http://a11y-eidas.dit.upm.es:8081 (accessed on 3 March 2021)	public
OAuth2.0 IdM	http://a11y-eidas.dit.upm.es:3000 (accessed on 3 March 2021)	public
Testing eIDAS node	http://a11y-eidas.dit.upm.es/EidasNode (accessed on 3 March 2021)	public
IdP proxy	http://a11y-eidas.dit.upm.es:8080 (accessed on 3 March 2021)	public
Attribute provider	http://a11y-eidas.dit.upm.es:5000 (accessed on 3 March 2021)	private
Demo IdP	http://a11y-eidas.dit.upm.es/IdP (accessed on 3 March 2021)	public

**Table 6:** User interfaces’ summary.

Interface	Description	Functional Attribute Condition
Interface 1	Information page	Other attribute combinations
	High contrast information page	Vision < 100
Interface 2	Information page adapted to cognition level	Cognition < 100
Interface 3	Voice user interface suggestion	Manipulation < 100, reach < 100
	Chatbot interface suggestion	
Interface 4	No images information page	Vision = 100
Interface 5	Information page with sign language video	Hearing = 100

**Table 7:** Users and attributes.

User	Attributes
**Visual impairments**
User 1 and User 2	vision = 100
User 3	vision = 30
User 4	vision = 75
User 5	vision = 20
**Hearing impairments**
User 6	hearing = 95
User 7	hearing = 30
User 8 to User 22	hearing = 100
**Aged people**
User 23	Reach = 40, vision = 20
User 24	No modifications
**No functional attributes**
User 25	No modifications
User 26	No modifications

**Table 8:** Survey results.

Question	Yes	No	Not Sure
1. I think that including my functional attributes provided by my national eID and authorized attribute providers when accessing web services will improve the usability and the quality of those services.	96.2%	3.8%	0%
2. I have used a fictitious eID that provides functional attributes corresponding to my capabilities and I think that using my citizen eID extended with those attributes facilitates the fulfillment of the objectives of the web service.	84%	8%	8%
3. I have used a fictitious eID that provides functional attributes corresponding to my capabilities and I think that using my citizen eID extended with those attributes improves the accessibility of the web service.	91.7%	8.3%	0%
4. After testing this pilot, I would like the inclusion of this functionality in other web services.	95.8%	0%	4.2%

**Table 9:** Comparison with related work. FIM, Federated Identity Management.

Ref.	User/Pass	FIM	Cross-Border	Extra Attrb.	eID/Token	A11y
Berbecaru et al. [23]		X	X	X	X	
Alonso et al. [24]		X	X	X	X	
Klobučar et al. [25]	X		X			
Stasis et al. [27]			X	X	X	
Buchmann et al. [30]			X		X	
García et al. [31]					X	X

## Data Availability

Not applicable.
